# Influence of Long-Distance Bicycle Riding on Serum/Urinary Biomarkers of Prostate Cancer

**DOI:** 10.3390/ijms17030377

**Published:** 2016-03-17

**Authors:** Zbynek Heger, Jaromir Gumulec, Ales Ondrak, Jan Skoda, Zdenek Zitka, Natalia Cernei, Michal Masarik, Ondrej Zitka, Vojtech Adam

**Affiliations:** 1 Department of Chemistry and Biochemistry, Mendel University in Brno, Zemedelska 1, CZ-613 00 Brno, Czech Republic; heger@mendelu.cz (Z.H.); cernei.natalia3@gmail.com (N.C.); zitkao@seznam.cz (O.Z.); 2 Central European Institute of Technology, Brno University of Technology, Technicka 3058/10, CZ-616 00 Brno, Czech Republic; masarik@med.muni.cz; 3 Department of Physiology, Faculty of Medicine, Masaryk University, Kamenice 5, CZ-625 00 Brno, Czech Republic; j.gumulec@med.muni.cz; 4 Faculty of Sports Studies, University Sports Centre, Masaryk University, Komenskeho namesti 2, CZ-662 43 Brno, Czech Republic; ondrak@fsps.muni.cz (A.O.); skoda@fsps.muni.cz (J.S.); zitka@fsps.muni.cz (Z.Z.)

**Keywords:** lactate, metabolism, physical exercise, prostate-specific antigen, sarcosine

## Abstract

Herein, we present a study focused on the determination of the influence of long-distance (53 km) bicycle riding on levels of chosen biochemical urinary and serum prostate cancer (PCa) biomarkers total prostate-specific antigen (tPSA), free PSA (fPSA) and sarcosine. Fourteen healthy participants with no evidence of prostate diseases, in the age range from 49–57 years with a median of 52 years, underwent physical exercise (mean race time of 150 ± 20 min, elevation increase of 472 m) and pre- and post-ride blood/urine sampling. It was found that bicycle riding resulted in elevated serum uric acid (*p* = 0.001, median 271.76 *vs.* 308.44 µmol/L pre- and post-ride, respectively), lactate (*p* = 0.01, median 2.98 *vs.* 4.8 mmol/L) and C-reactive protein (*p* = 0.01, 0.0–0.01 mg/L). It is noteworthy that our work supports the studies demonstrating an increased PSA after mechanical manipulation of the prostate. The subjects exhibited either significantly higher post-ride tPSA (*p* = 0.002, median 0.69 *vs.* 1.1 ng/mL pre- and post-ride, respectively) and fPSA (*p* = 0.028, median 0.25 *vs.* 0.35 ng/mL). Contrary to that, sarcosine levels were not significantly affected by physical exercise (*p* = 0.20, median 1.64 *vs.* 1.92 µmol/mL for serum sarcosine, and *p* = 0.15, median 0.02 µmol/mmol of creatinine *vs.* 0.01 µmol/mmol of creatinine for urinary sarcosine). Taken together, our pilot study provides the first evidence that the potential biomarker of PCa—sarcosine does not have a drawback by means of a bicycle riding-induced false positivity, as was shown in the case of PSA.

## 1. Introduction

Mechanical stimulation of prostate is a generally-accepted interfering factor for the detection of prostate-specific antigen (PSA) level [1], but the available data are controversial. Long-distance biking is an excellent model to study the combined effects of mechanical prostate stimulation by bicycle riding and strenuous endurance exercise [2]. Mejak and coworkers demonstrated that cycling causes a significant increase in total PSA level when measured within 5 min post-cycling [3], whereas similar results were also achieved in other studies [4,5,6,7]. On the other hand, other researchers report no changes in PSA level following cycling [8,9,10], showing the controversy of these types of studies. The methodologies however varied in numerous parameters, including the age, timing of the blood sampling and the duration and intensity of physical exercise; thus, the comparison is considerably hindered. PSA might be also increased as a consequence of a digital rectal examination, transrectal ultrasound or any kind of biopsy [11]. Hence, it is obvious that PSA concentrations can be altered by various physical events. Considering the fact that the standard PSA cut-off (4 ng/mL) exhibits relatively low sensitivity [12] and specificity [13], novel biomarkers of prostate cancer (PCa) are required. One of them, sarcosine, is currently widely discussed as a potential biomarker for the early stages of PCa [14,15]. It is a non-proteinogenic amino acid occurring as an intermediate product in the glycine metabolic pathway [16]. Since its first mention in 2009 [17], sarcosine has been investigated as an important oncometabolite. As the activation of metabolism during intense, prolonged, muscular activity is limited by the properties of the transport functions of blood, it can be hypothesized that sarcosine metabolism might be impaired by such a kind of physical exercise. Nevertheless, to the best of our knowledge, there is no work available regarding whether the urinary and serum levels of sarcosine are influenced by long-distance bicycle riding.

Our current objective was therefore to determine the influence of long-distance bicycle riding on the levels of serum free PSA (fPSA), total PSA (tPSA) and sarcosine. Moreover, to get the maximum amount of information, both matrices were subjected to biochemical analyses, including amino acid patterning. Overall, the disparities in biochemical biomarkers in pre- and post-ride specimens are highlighted.

## 2. Results

### 2.1. Comparison of Pre- and Post-Ride Anthropometric Characteristics

The tested cohort (*n* = 14) was exposed to long-distance bicycle riding (53 km) with a total ascent of 472 m. The mean race time was 150 ± 20 min. Wilcoxon matched pairs test revealed a significant difference in systolic pressure (Table 1). Although % fat and % muscles are indicated as statistically significant, the clinical significance is groundless (compare the medians in Table 1).

**Figure 1:**
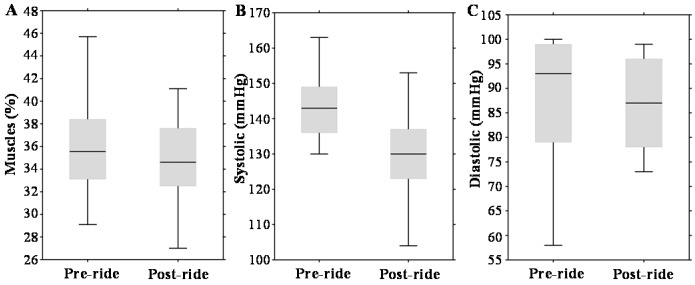
Effect of physical exercise on anthropometric parameters, expressed as box plots for: (**A**) total muscles; (**B**) systolic pressure; and (**C**) diastolic pressure. The middle lines in the figures indicate the median values.

Figure 1A–C illustrates that physical exercise had a significant impact on body muscle content (about a 3% decrease, *p* = 0.01) and systolic blood pressure (about a 10% decrease, *p* = 0.005). Diastolic pressure was not altered significantly.

### 2.2. Comparison of Pre- and Post-Ride Serum and Urinary Biochemical Markers

After venipuncture from the antecubital vein, pre- and post-ride serum biochemical markers were evaluated. There were no significant differences between pre- and post-ride triglycerides, alkaline phosphatase (ALP), alanine transaminase (ALT), aspartate transaminase (AST), γ glutamyltransferase (GMT), cholesterol, glucose, total protein and creatinine (Table 2). A slight increase was found in serum bilirubin (*p* = 0.60) (median 7.35 µmol/L (range 6.38–10.33 µmol/L) *vs.* median 9.8 µmol/L (range 7.92–10.93 µmol/L)). Testosterone levels were increased in the pre-ride serum specimens (*p* = 0.727) (median 6.9 nmol/L (range 5.1–11.7 nmol/L) *vs.* median 8.5 nmol/L (range 5.9–9.4 nmol/L)).

**Figure 2:**
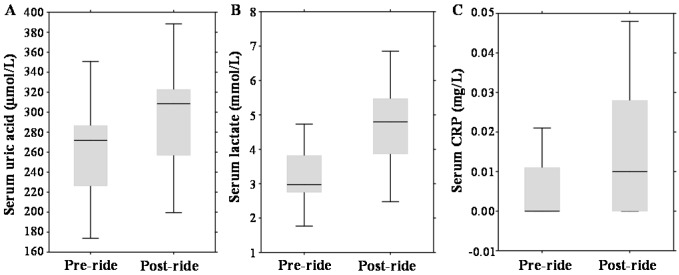
Effect of physical exercise on biochemical parameters, expressed as box plots for: (**A**) serum uric acid; (**B**) serum lactate; and (**C**) serum C-reactive protein (CRP). The middle lines in the figures indicate the median values.

Regarding other serum parameters, post-ride measurements revealed significantly elevated concentrations of serum uric acid by 13% (*p* = 0.001) (median 271.76 µmol/L (range 226.28–286.6 µmol/L) *vs.* median 308.44 µmol/L (range 256.8–322.69 µmol/L)); serum lactate increased by 61%, (*p* = 0.01) (median 2.98 mmol/L (range 2.75–3.82 mmol/L) *vs.* median 4.8 mmol/L (range 3.87–5.47 mmol/L)); and serum C-reactive protein (CRP) increased from undetectable values (*p* = 0.01) (median 0.0 mg/L (range 0.0–0.01 mg/L) *vs.* median 0.01 mg/L (range 0.0–0.03 mg/L)); which is illustrated in Figure 2A–C.

Pre- and post-ride testing of urinary specimens, including determination of amino acid patterns, revealed that physical exercise resulted in a decrease of pH (median 5.7 (range 5.1–6.0) *vs.* 5.1 (4.8–5.5)). A comparison of other urinary parameters showed no significant differences (Table 3).

### 2.3. Evaluation of Pre- and Post-Ride Levels of Prostate Cancer (PCa) Biomarkers

**Figure 3:**
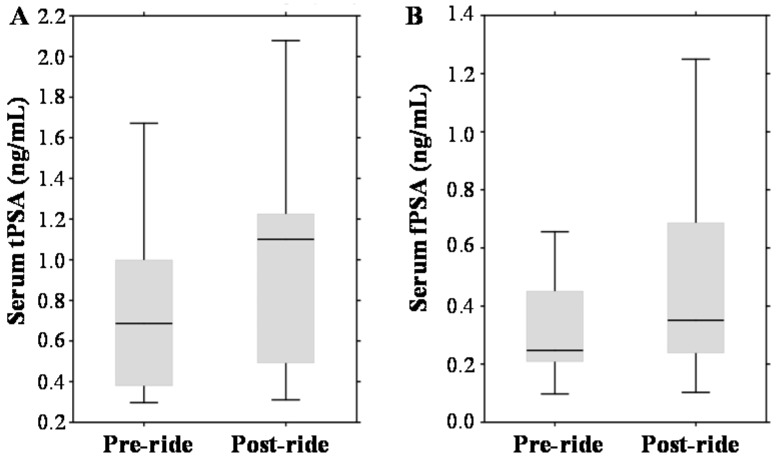

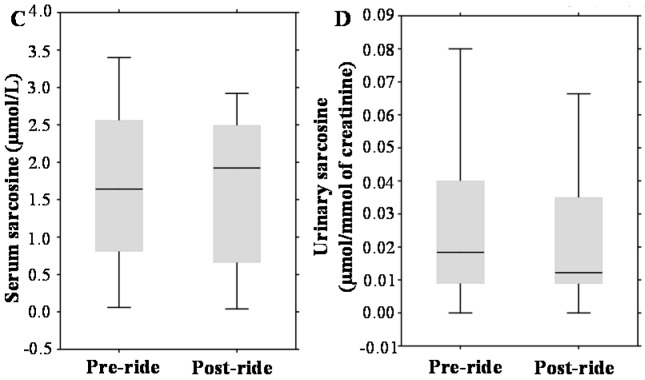
Effect of physical exercise on biomarkers of prostate cancer (PCa), expressed as box plots for: (**A**) serum total prostate-specific antigen (tPSA); (**B**) serum free PSA (fPSA); (**C**) serum sarcosine; and (**D**) urinary sarcosine. The middle lines in the figures indicate the median values.

In the pre-ride testing, the tPSA median value was 0.69 ng/mL with a range 0.38–1.0 ng/mL (Figure 3A). Considering the standard PSA cut-off (4.0 ng/mL), no participants with elevated PSA were involved into the study cohort. Post-ride measurements revealed significant elevation to a median value of 1.1 ng/mL (range 0.49–1.23 ng/mL). Using the standard PSA cut-off, all participants remained below the limit values (even though age-based normal ranges have been considered). PSA exhibited also a well-documented positive correlation with the age of participants (*r* = 0.46 at *p* = 0.17), their CRP (*r* = 0.60 at *p* = 0.001) and serum creatinine (*r* = 0.61 at *p* = 0.001). A similar exercise-dependent growing trend was observed in the case of serum fPSA (median 0.25 ng/mL (range 0.21–0.45 ng/mL) *vs.* median 0.35 ng/mL (range 0.24–0.69 ng/mL)), which is shown in Figure 3B.

To the contrary, Figure 3C,D demonstrates that the serum and urinary sarcosine concentrations were not affected by bicycle riding (median 1.64 µmol/mL (range 0.81–2.56 µmol/mL *vs.* median 1.92 µmol/mL (range 0.66–2.49 µmol/mL); and median 0.02 µmol/mmol of creatinine (range 0.01–0.04 µmol/mmol of creatinine) *vs.* median 0.01 µmol/mmol of creatinine (range 0.01–0.04 µmol/mmol of creatinine)).

## 3. Discussion

In the present work, we investigated the influence of cycling on biochemical markers in serum and urinary specimens of healthy subjects with no evidence of a clinically-significant benign prostatic hyperplasia or malignancy. The primary purpose was to determine whether physical exercise affects the concentrations of routinely-used PCa biomarkers tPSA and fPSA and the serum and urinary levels of sarcosine, a potential biomarker of PCa [17].

### 3.1. Evaluation of Pre- and Post-Ride Anthropometric and Serum Biochemical Parameters

Primarily, we observed expected changes in anthropometric parameters in response to exercise. A decrease in detected muscle content is connected to dehydration, which is most commonly seen after strenuous exercise, in which heavy sweating has occurred [18]. Exercise also increases blood flow and vascular perfusion in skeletal muscles, which can result in a decrease in bioimpedance and muscle resistivity. This phenomenon can further affect the concentration of minerals in serum or disturb the functions of the cardiovascular system [19]. It can be stated that there is a general agreement that the systolic blood pressure is increased after aerobic exercise; nevertheless, diastolic blood pressure exhibits negligible changes only [20].

Lactate accumulation has been for a long time linked with disturbed sports performance, connected with the hypothesis about lactate acidosis [21]. Nevertheless, the lactate-shuttle mechanism enables the use of lactate as an energetic substrate in both type I and type II skeletal muscles, providing them for physical exercise [22]; thus, lactate is considered as a well-established indicator of fatigue more than as its cause. Based on the above-mentioned facts, the post-ride elevation of lactate level confirms that the level of physical exercise of the cyclist was sufficient.

Looking at the effects of bicycle riding on the determined biochemical markers, we found that levels of the main androgen steroid hormone testosterone were increased. It is well known that testosterone levels vary after an intensive and prolonged physical exercise [23], and it was found that strenuous running or weight-lifting resulted in an increase of testosterone levels [24]. On the other hand, Saka and coworkers showed that rigorous cycling decreased testosterone levels (*p* = 0.001); nevertheless, why serum testosterone is reduced is not clear [9]. They hypothesized that hormone production can be affected by constant abrasion of the testicles, leading to elevation of intrascrotal temperature or testicular microtrauma, and indeed, all of these factors impact the testicular functions [25]. Contrary to that, our results show a post-ride elevation of testosterone, which can be partly a result of a transient hemoconcentration of circulating testosterone, reduced metabolic clearance and/or a possible hormone-mediated increase of testicular production [26].

Further, we detected modest differences in pre- and post-ride bilirubin values of cyclists. Although we did not investigate specific mechanisms involved in this phenomenon, the literature suggests that exercise may increase the activity of heme-oxygenase-1, which converts biliverdin to bilirubin [27]. Since bilirubin can be produced through its pre-cursor heme [28], another plausible mechanism of bilirubin elevation is the stimulation of heme catabolism through an exercise-induced hemolysis and subsequent increased heme bio-availability, promoting the increased bilirubin concentrations. There is a major lack of studies concerning the effects of aerobic exercise on the bilirubin levels; however, we can find the support in a study by Swift and coworkers [29], who concluded the possible beneficial effect on the decrease of cardiovascular risk through an increase in bilirubin concentrations.

Uric acid has a pivotal influence on vascular control by elevating oxidative stress (O_2_^−^ production) and NO-scavenging, which lead to vasodilatation [30]. Our results demonstrate increased post-ride serum uric acid. It is worth noting that all participants carried out regular aerobic exercise, which increases the activity of xanthine oxidase, contributing to the oxidative stress [31]. This enzyme is connected in the oxidation of hypoxanthine to xanthine and subsequently to uric acid after degradation of adenosine triphosphate (ATP) to adenosine monophosphate (AMP) and inosine-5′-monophosphate (IMP). Thus, the post-ride elevation of uric acid (the end product of purine nucleotide catabolism) is a result of physical exercise-induced purine degradation. Taken together, although physical activity improves cardiovascular risk, on the other hand, the impact of immediate uric acid formation on hyperuricemia or chronic gout is still not yet elucidated.

Our analyses revealed very low levels of CRP in pre-ride and post-ride serum specimens. It was found that bicycle riding resulted in a modest elevation of CRP; however, its amount was still very low and classified as a normal systemic CRP level [32]. The available literature provides contradictory results on CRP levels post-physical exercise, since both an increase [33] and a decrease [34] have already been described. This phenomenon is likely due to the various types of activities tested in studies; however, it seems that CRP elevation is mostly associated with an exercise-induced inflammatory sequelae and is more significant during activities that are longer or more aerobically demanding, such as bicycle riding for longer distances.

### 3.2. Evaluation of Pre- and Post-Ride Urinary Biochemical Parameters

Analyses of urinary specimens confirmed that physical exercise causes urinary pH to shift towards acidity [35], owing to induced metabolic acidosis. Further, we focused on the determination of urinary amino acid patterns. These can precisely reflect the metabolomic status of an organism, as was shown in the case of various types of cancers [36]; however, within the meaning of our tested cohort, we did not observe any significant changes. Hence, it can be stated that changes in amino acids are present mostly as a result of metabolic alterations [37] other than strenuous physical exercise and, instead of plasma amino acids [38], urinary amino acids are stable in their pre- and post-ride content.

### 3.3. The Effect of Physical Exercise on PCa Biomarkers

In the examination of PCa biomarkers, the study design resulted in an increase of both analyzed forms of PSA (tPSA; fPSA). By testing participants of the same age (about ≥50 years old), we mention two essential studies based on measurements of PSA in men after bicycle riding. Although the level of physical activity differed modestly, both studies describe an elevation in post-ride specimens [3,6] and, thus, are consistent with our findings. It is noteworthy that Kindermann *et al.* showed that one hour cycle ergometer activity increased not only tPSA, but also fPSA [5].

**Figure 4:**
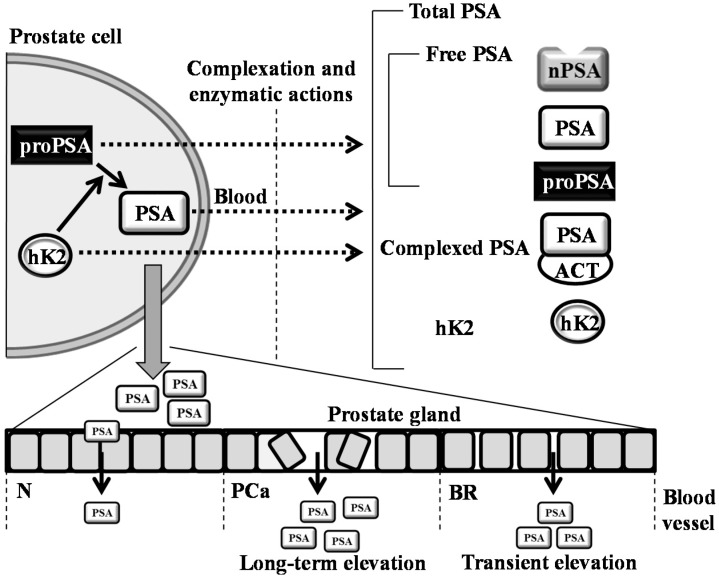
Schematic depiction of the formation of PSA variants and kallikrein-related peptidase 2 (hK2). hK2 proteolytic activity probably removes propeptides from PSA, forming its mature, catalytic form. Secreted PSA is complexed or enzymatically inactivated to form free PSA forms (nicked PSA (nPSA), proenzyme form of PSA (proPSA) and intact PSA) and complexed PSA (PSA complex with ACT (1-antichymotrypsin)). In normal conditions (N), cells in the prostate are healthy and organized in a tight pattern. Hence, only a small amount of PSA leaks into the bloodstream. In prostate cancer (PCa), cells are disorganized, and the layers between the prostate and bloodstream become disrupted. Thus, more PSA is leaked into the blood. During bicycle riding (BR), cells are likely irritated and forming the pores for the transient leakage of PSA into blood. This phenomenon can significantly affect the clinical performance of PSA. The dotted arrows represent the transition of molecules from the intracellular to the extracellular regions.

Contrary to that, our study concurs with the data obtained in other studies. For instance, Saka and colleagues determined that a 300-km bicycle ride does not impact the performance of tPSA and fPSA [9]; nevertheless, the study design is not comparable to our study, due to the tested cohort. In contrast to our cohort, they involved athletes and student volunteers with an average age of 22.4 and 24.4 years, respectively. Other studies with contradictory results can be found [2,8,10]; nevertheless, a closer look reveals that their methodologies varied in many parameters, including the age, timing of the blood sampling and the duration and intensity of physical exercise; thus, the comparison is considerably hindered. Overall, it can be stated that similarly to a disorganization of prostate cells during PCa development, physical activity can irritate the cells to form transient pores for the leakage of PSA into blood (Figure 4).

The stability of a biomarker belongs to its most fundamental properties. By means of physical exercise, both urinary and serum sarcosine exhibited no differences among pre- and post-ride sampling. The rationale of testing sarcosine is its possible utilization as an auxiliary diagnostic tool for PCa diagnostics [17]. Contrary to PSA, which is a secretory protein, sarcosine is a common intermediate metabolite and by-product in glycine synthesis and degradation through the activity of glycine-*N*-methyltransferase (GNMT) [16]. Thus, it is expected that the sarcosine amount is highly dependent on the action of GNMT, which is encoded by tumor-susceptible gene GNMT [39]. To the best of our knowledge, there is a lack of evidence about the effects of physical exercise on GNMT enzymatic activity; however, our results indirectly illustrate no significant changes in the degradation of glycine (Table 3) and the elevation of sarcosine. Obviously, the impact on other biochemical markers does not interfere with the sarcosine urinary/serum levels, either. Hence, sarcosine seems to be applicable in prostate examinations, especially in the cases where PSA is expected to be undesirably influenced (per-rectal examination, bladder catheterization, sexual activity or bicycle riding).

## 4. Materials and Methods

### 4.1. Chemical Compounds

All standards and other chemicals were obtained from Sigma-Aldrich (St. Louis, MO, USA) in ACS purity, unless noted otherwise.

### 4.2. Volunteers, Physical Exercise and Sample Collection

**Figure 5:**
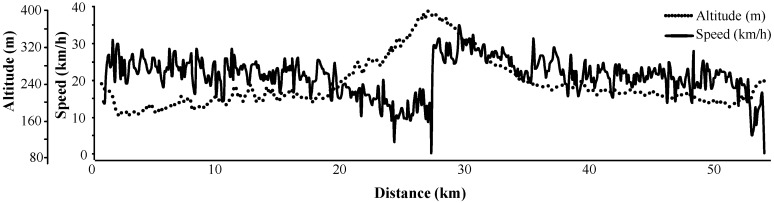
Profile of the physical exercise (bicycle riding) performed to evaluate the differences between anthropometric and biochemical parameters.

The cohort consisted of 14 male cyclists with no evidence of a PCa. The age ranged between 49 and 57 years with a median age of 52 years and a median height of 182.5 cm (interquartile range 179–185 cm). The planned route was 53 km long with a total elevation increase of 472 m, whereas the recommended speed was 20 km/h (the profile of the ride is illustrated in Figure 5). Participants with signed informed consent were advised to abstain from physical activity, including bicycle riding and sexual intercourse, for 24 h until their pre-cycling blood/urinary tests were taken, and they were on a diet for 24 h prior to the ride (no protein-rich and energy-rich food, no protein supplements, no alcohol). Participants were asked to sit without changing posture and with minimal movement during the ride. They had their pre-ride and post-ride anthropometric measurements and blood (venipuncture from antecubital vein)/urine sampling within 10 min before starting the ride and within 10 min after finishing the ride.

### 4.3. Anthropometric Measurements

Anthropometric measurements were carried out using the bioimpedance measurements on the body composition monitor and scale, Omron HBF-514C (Omron, Kyoto, Japan). The determined parameters were: fat, muscle, body mass index (BMI) and weight. Before and after the ride, we monitored the blood pressure (digital tonometer Tensoval Duo Control, Hartmann Rico, Veverska Bityska, Czech Republic) and temperature (non-contact infrared forehead thermometer BC 07, Body Comfort, Horomerice, Czech Republic) of the participants.

### 4.4. Biochemical Analyses of Serum Specimens

tPSA, fPSA and testosterone were determined using commercial immunoenzymometric assays (ST AIA-PACK PSA II, ST AIA-PACK ucPSA II and ST AIA-PACK Testosterone, Tosoh Bioscience, Tokyo, Japan) on the immunoanalyzer AIA 600 II (Tosoh Bioscience) according to the manufacturer’s instructions. Other biochemical parameters were determined using the commercial kits on the automated spectrophotometer BS-400 (Mindray, Shenzhen, China). The utilized kits were: glucose assay, uric acid assay, lactate assay, triglycerides assay, alkaline phosphatase (ALP) assay, alanine transaminase (ALT) activity assay, aspartate transaminase (AST) activity assay, γ glutamyltransferase (GMT) assay, cholesterol assay, bilirubin total assay, creatinine assay, C-reactive protein (CRP) assay purchased from Greiner (Stuttgart, Germany) and pyrogallol red protein assay (Skalab, Svitavy, Czech Republic).

### 4.5. Biochemical Analyses of Urinary Specimens

The pH of urine was determined using the pH meter WTW inoLab (Weilheim, Germany). Na^+^, K^+^ and Cl^−^ ions were determined using ion-selective electrodes, where the AgCl electrode was employed as a reference one (Metrohm, Herissau, Switzerland). Total protein content and creatinine were detected using the commercial kits (pyrogallol red protein assay, Skalab and creatinine kit, Greiner) on the automated spectrophotometer BS-400 (Mindray), according to the manufacturer’s instructions. Urinary amino acid patterns were obtained after an acidic hydrolysis using ion-exchange chromatography (IEC, AAA-400, Ingos, Prague, Czech Republic) with post-column derivatization by ninhydrin and the absorbance detector in the visible range (IEC-VIS), according to our previous study [40].

### 4.6. Preparation of Serum and Urinary Specimens for Sarcosine Determination

Urine was prepared by simple evaporation with subsequent dilution with dilution buffer according to Heger *et al.* [40]. Sera (250 μL) were deproteined using 250 μL of 10% trifluoroacetic acid (*v*/*v*) and centrifuged using the Microcentrifuge 5417R (Eppendorf AG, Hamburg, Germany) under 25,000× *g* at 4 °C for 15 min. Centrifugation of the supernatant was repeated once to remove the residues of the precipitated proteins. The resulting supernatant was analyzed on IEC-VIS, whereas the analyses were carried out following the conditions from our previous study [41].

### 4.7. Statistical Analysis

The results were analyzed using the Wilcoxon matched pairs test. Correlation between variables was performed using Pearson correlation. Statistical processing was carried out by Software Statistica 12 (StatSoft, Tulsa, OK, USA). Unless noted otherwise, a *p*-level < 0.05 was considered significant.

## 5. Conclusions

Our findings suggest that bicycle riding is able to significantly elevate the tPSA and fPSA levels determined in post-ride specimens, sampled 10 min after completion of the ride. We define this increase as clinically significant, since this impact can result in false positive results and inaccurate indications for biopsy in men having naturally-elevated PSA. Hence, we absolutely agree with the up-to-date available literature recommending abstaining for activities, such as bicycle riding, for 24–48 h. Contrary to that, both analyzed specimens exhibited constant levels of sarcosine. Hence, we postulate that this low-mass metabolite might be a helpful auxiliary tool in PCa diagnostics with relatively constant amounts, when compared to other biochemical markers, including PSA. Indeed, our pilot study has a few drawbacks. One of them is the relatively small cohort of tested subjects, which however provided consistent data comparable to studies with a similar design. Our tested cohort also did not include subjects with baseline PSA close or above 4 ng/mL nor subjects with confirmed prostate malignancies. This phenomenon however made our cohort highly homogenous for the entire statistical analyses. Regarding the fact that PSA levels can be affected by various mechanical manipulations of prostate, including ejaculation, sarcosine can be considered as a steady biomarker, complementary to other clinical tests.

## Figures and Tables

**Table 1:** Anthropometric characteristics of the cohort; results of the Wilcoxon matched pairs test.

Parameter	Before (Median, Interquartile Range)	After (Median, Interquartile Range)	*p*-Value
Muscles (%)	35.55 (33.1–38.4)	34.6 (32.5–37.6)	0.01
Fat (%)	22.5 (16.4–25.5)	22.75 (18.4–27.3)	0.01
Temperature (°C)	36.4 (36.2–36.6)	36.1 (36–36.3)	0.08
Body mass index (BMI) (kg/m^2^)	25.9 (23.6–27.7)	25.9 (23.8–27.4)	0.10
Systolic pressure (mmHg)	143 (136–149)	130 (123–137)	0.01
Diastolic pressure (mmHg)	93 (79–99)	87 (78–96)	0.10

**Table 2:** Serum markers of tested cohorts; results of the Wilcoxon matched pairs test.

Parameter	Before (Median, Interquartile Range)	After (Median, Interquartile Range)	*p*-Value
Testosterone (nmol/L)	6.9 (5.1–11.7)	8.5 (5.9–9.4)	0.727
Triglycerides (mmol/L)	1.7 (1.35–2.14)	1.6 (1.32–1.97)	0.81
ALP (µkat/L)	0.53 (0.5–1.29)	0.87 (0.46–1.06)	0.75
ALT (µkat/L)	0.54 (0.47–0.64)	0.52 (0.49–0.56)	0.31
AST (µkat/L)	0.43 (0.41–0.5)	0.48 (0.43–0.51)	0.13
GMT (µkat/L)	0.46 (0.38–0.69)	0.45 (0.34–0.75)	0.55
Cholesterol (mmol/L)	5.75 (5.34–6.21)	5.9 (5.28–6.34)	0.14
Bilirubin (µmol/L)	7.35 (6.38–10.33)	9.8 (7.92–10.93)	0.60
Glucose (mmol/L)	4.93 (4.56–5.17)	4.48 (4.38–4.99)	0.20
Total protein (g/L)	65.48 (62.26–66.36)	65.71 (64.04–67.2)	0.31
Creatinine (µmol/L)	100.74 (96.9–103.9)	103.21 (95.02–109.31)	0.42

ALP: alkaline phosphatase; ALT: alanine transaminase; AST: aspartate transaminase; GMT: γ glutamyltransferase.

**Table 3:** Urinary markers of tested cohorts; results of the Wilcoxon matched pairs test.

Parameter	Before (Median, Interquartile Range)	After (Median, Interquartile Range)	*p*-Value
pH	5.7 (5.1–6)	5.1 (4.8–5.5)	0.04
Potassium (mmol/mmol) *	32 (23–38)	28.5 (17–43)	0.75
Chlorides (mmol/mmol) *	244.5 (187–294)	204.5 (137–247)	0.08
Sodium (mmol/mmol) *	118 (79–178)	113 (56–164)	0.51
Total protein (g/L)	16.65 (12–32.1)	17.8 (11.5–25)	0.27
Creatinine (mmol/L)	6.96 (5.2–8.29)	6.59 (3.64–8.7)	0.78
Valine (µmol/mmol) *	13.35 (7.9–24.4)	13.2 (3.8–25.3)	0.62
Tyrosine (µmol/mmol) *	9.25 (3.8–17.3)	6.55 (4.1–14.6)	0.40
Threonine (µmol/mmol) *	10.4 (1.8–19.4)	9.55 (5.8–19.5)	0.59
Serine (µmol/mmol) *	6.95 (3.5–11.6)	6.45 (3.6–9.5)	0.30
Proline (µmol/mmol) *	4.6 (3.3–9.7)	4.75 (2.8–9.8)	0.83
Phenylalanine (µmol/mmol) *	7.95 (6.2–15.2)	8.35 (5.1–16.6)	1.00
Methionine (µmol/mmol) *	5.15 (2.4–10.4)	5.25 (2.6–11.3)	0.64
Lysine (µmol/mmol) *	49.6 (38.6–85.6)	51.5 (34.1–130.1)	0.20
Leucine (µmol/mmol) *	4.05 (0.9–9.8)	2 (0.8–3.5)	0.11
Isoleucine (µmol/mmol) *	4.6 (1–9.5)	5.35 (2.6–7.7)	0.97
Histidine (µmol/mmol) *	14.75 (3.6–50.6)	11.5 (7.2–44.8)	0.36
Glycine (µmol/mmol) *	6.3 (3–13)	7.35 (3.9–11.1)	0.36
Glutamic acid (µmol/mmol) *	3.45 (1.1–6)	3.5 (1.3–7.3)	0.81
Cysteine (µmol/mmol) *	4.2 (2.8–10.4)	4.45 (1.8–5.3)	0.64
Aspartic acid (µmol/mmol) *	27.5 (15.6–64.2)	33 (20.1–66.7)	0.55
Arginine (µmol/mmol) *	39.55 (16.3–61.7)	30.3 (18.6–38.1)	0.06
Alanine (µmol/mmol) *	3.55 (1.9–6.3)	4.25 (1.7–7.2)	0.25

* Values are normalized to mmol of creatinine.

## Abbreviations

The following abbreviations are used in this manuscript:

tPSA	Total prostate specific antigen
fPSA	Free prostate specific antigen
nPSA	Nicked PSA
PSA	Prostate specific antigen
ALP	Alkaline phosphatase
ALT	Alanine transaminase
AST	Aspartate transaminase
GMT	Gamma glutamyltransferase
CRP	C-reactive protein
ATP	Adenosine triphosphate
AMP	Adenosine monophosphate
IMP	Inosine-5′-monophosphate
IEC-VIS	Ion-exchange chromatography with detector in visible range
hK2	Kallikrein-related peptidase 2
ACT	1-antichymotrypsin
BR	Bicycle riding
